# “Esketamine” in Borderline Personality Disorder: A Look Beyond Suicidality

**DOI:** 10.7759/cureus.24632

**Published:** 2022-04-30

**Authors:** Neethu K Nandan, Puneet K Soni, Ajay Parsaik, Aqeel Hashmi

**Affiliations:** 1 Psychiatry, All India Institute of Medical Sciences, Raipur, Raipur, IND; 2 Psychiatry, All India Institute of Medical Sciences, Jodhpur, Jodhpur, IND; 3 Psychiatry, Marshfield Clinic Health System, Marshfield, USA; 4 Psychiatry, Westpark Springs Hospital, Richmond, USA

**Keywords:** suicidal ideations, treatment-resistant major depressive disorder, borderline personality disorder, ketamine, esketamine

## Abstract

Borderline personality disorder (BPD) is an extremely disabling condition that affects almost every dimension of a patient’s life. The S-enantiomer of ketamine (esketamine) was approved by the Food and Drug Administration (FDA) in 2019 in conjunction with an oral antidepressant for the management of treatment-resistant depression (TRD) in adults. Our patient is a 27-year-old female with a long-standing diagnosis of BPD and treatment-resistant major depressive disorder (MDD) who presented to a tertiary care hospital after a baleful suicide attempt. As per treatment guidelines, “esketamine” intranasal spray in conjunction with citalopram 20 mg was started in the outpatient setting at a dose of 56 mg twice weekly for four weeks, followed by 56 mg once weekly, which was further titrated to 84 mg once weekly. Two years into treatment, the patient and her mother report around 70% improvement in her depression and anxiety with around 80% improvement in her behavioral symptoms. Esketamine’s potential action on patients with BPD can be partially explained by its very well-documented effect on the glutamate receptor antagonism. Additionally, patients with stress-induced suicidal ideations (SI), which are seen in borderline patients, are better responsive to ketamine. In conclusion, we recommend a trial of intranasal esketamine in patients with BPD with treatment-resistant MDD and frequent episodes of self-harm. Treatment with esketamine could potentially reduce the number of emergency room visits for impulsive suicide attempts and help reduce the life burden of BPD and its impact on family members.

## Introduction

Borderline personality disorder (BPD) is an extremely disabling condition that affects almost every dimension of a patient’s life. It can present with a multitude of symptoms, including affective instability, impulsivity, unstable self-image, interpersonal relationship issues, and self-injurious behaviors. Borderline personality disorder is often associated with comorbid conditions such as eating disorders, mood disorders, and substance use disorders [1]. The prevalence of BPD and major depressive disorder (MDD) are about 5.9% and 8%, respectively, but up to 80% of patients with BPD experience one or more episodes of MDD in their lifetime [1]. Conversely, 10%-30% of patients with MDD have co-occurring BPD. The disorder was initially conceptualized as bordering between psychosis and neurosis according to psychoanalytic theories [2]. However, BPD is a complex disorder, and a converging body of scientific evidence indicates that patients suffer from impaired functional connectivity between various brain regions involved in emotional processing, affect, and impulsivity, such as the amygdala, insula, posterior cingulate cortex, hippocampus, anterior cingulate cortex, and prefrontal regulatory regions, with different endophenotypes of the disorder [3-5]. From a clinical standpoint, BPD can be very difficult to treat. Although psychotherapy is the mainstay of the treatment, several pharmacotherapeutic agents have been considered, such as antidepressants, mood stabilizers, and antipsychotics, with varying degrees of response. Patients with BPD in acute crisis may not be amenable to therapy, especially if they are acutely suicidal [6].

Racemic ketamine was first introduced into clinical practice in the 1960s as an invaluable anesthetic. However, its use in the management of treatment-resistant depression (TRD) is a much more recent addition. The S-enantiomer of ketamine (esketamine) was approved by the Food and Drug Administration (FDA) in 2019 in conjunction with an oral antidepressant for the management of TRD in adults [7]. Although the ketamine intravenous infusion was argued to have better efficacy than esketamine, the method of administration (intranasal) made esketamine easier to use than the former [8]. Various studies point toward ketamine and its enantiomer’s effectiveness in treating symptoms and suicidality in patients with depression [9]. However, most of these studies included patients with mood disorders only, especially in patients with treatment-resistant depression, resulting in data that does not necessarily reflect the complete picture of cases that come to the emergency room in acute suicidal crisis [10].

In this case report, we explore the case of a 27-year-old female with a diagnosis of BPD with treatment-resistant depression and chronic suicidal ideations (SI) with acute exacerbation, who is being successfully treated with intranasal esketamine with positive results.

## Case presentation

The patient is a 27-year-old female with a long-standing diagnosis of BPD and treatment-resistant major depressive disorder (MDD) who presented to a tertiary care hospital after a baleful suicide attempt. Her social history includes adoption at birth and a history of substance abuse from her biological mother. There is no information regarding her biological father, and her biological family did not have any contact with the patient after adoption. Her developmental milestones were eventually achieved on time. She was diagnosed with MDD at the age of seven years and has used numerous medications ever since, with minimal improvement. By the age of 8-9 years, she displayed symptoms suggestive of an evolving borderline personality, characterized by repeated self-harm, mood swings, and anger outbursts. Because of the family’s burnout from these behaviors, she was physically abused many times. She had difficulty making and maintaining friends. She also had body image issues with diet restriction and alternating episodes of binge eating and self-induced vomiting. She was average in studies by this age where therapy was started focusing on her borderline symptoms. By the age of 15, she had exposure to various substances, including alcohol, cannabis, methamphetamine, cocaine, and nicotine. There were several episodes of impulsive sexual encounters and shopping. Her adopted mother tried to stop giving her money to cut down on her behaviors. However, this led her to prostitution as a means of obtaining drugs. Eventually, she was placed in a rehabilitation facility for six months. Thereafter, her romantic relationships were often unstable, and she would easily get overwhelmed and would engage in self-harm in the form of cutting her wrists and thighs with sharps or burning herself with a hair straightener. Even seemingly neutral stimuli would tick her off, and she would engage in impulsive self-harm or throw a fit of rage. These behaviors often affected her work environment, and she had difficulty maintaining a stable job. She attempted multiple suicidal and non-suicidal self-harm episodes. Most of her suicidal attempts occurred in the presence of people and were impulsive in nature. The patient reports that her attempts were not well thought out or planned.

The current suicidal attempt occurred after an altercation with her boyfriend with whom she had a child. She had locked herself in a room and impulsively ingested almost one hundred 1 mg alprazolam tablets in an attempt to kill herself. On presentation to the emergency room, the patient was intubated, and supportive management was commenced. Her urine drug test was positive for benzodiazepines. Her routine investigations, such as electrocardiogram (ECG), liver function test, renal function test, thyroid function test, and complete blood count, were normal on presentation. In the emergency room, she remained agitated toward staff and family. During the hospital course, she reported a long-standing feeling of sadness, hopelessness, helplessness, and continuing suicidal ideations. She tried to jump from the second floor of the hospital after a conversation with the medical staff that made her agitated. To help prevent further self-harm, she was restrained and placed under one-to-one observation. After medical stabilization, she was transferred to an inpatient psychiatric hospital for the management of her mood disorder and borderline personality disorder.

A review of treatment history revealed previous failed adequate trials of sertraline, fluoxetine, escitalopram, mirtazapine, venlafaxine, bupropion, lamotrigine, quetiapine, aripiprazole, lithium, and divalproex, alone or in combination. She had already undergone various psychotherapies in the form of dialectical behavioral therapy, cognitive behavioral therapy, group therapy, mindfulness-based therapy, etc., but the level of motivation and engagement in the therapy could not be commented. None of these treatments provided satisfactory symptom relief with respect to her depression, and she was not able to use her coping skills during emotional crises, leading to multiple past emergency room visits. When considered stable for outpatient treatment, she was discharged with recommendations for frequent outpatient psychiatric treatment. Esketamine was considered for appropriate treatment and maintenance as the literature shows immediate and sustained effects of esketamine treatment-resistant depression [9]. Her substance use history was carefully probed, and it was established that she had never been on any substance in a dependence pattern other than nicotine. After a discussion of the potential risks and benefits with the patient, a decision was made to treat the patient with intranasal esketamine targeting her treatment-resistant depression with current suicidal ideation.

**Figure 1:**
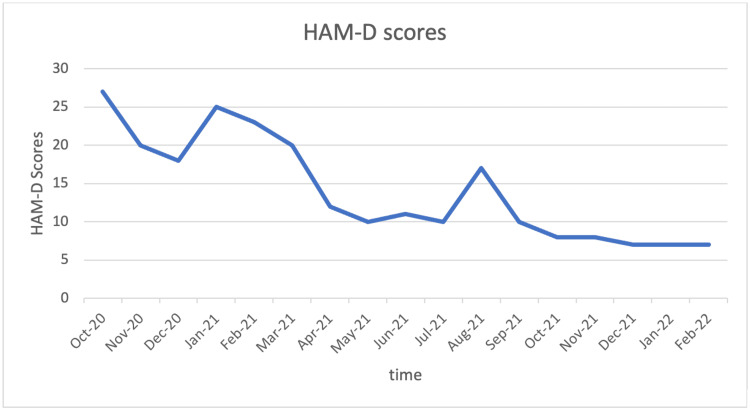
Regression in HAM-D score on treatment with esketamine over time

Esketamine intranasal spray was started in the outpatient setting at a dose of 56 mg twice weekly for four weeks, followed by 56 mg once weekly, which was further titrated to 84 mg once weekly. As per treatment guidelines, esketamine was administered in conjunction with citalopram 20 mg daily. She was also prescribed Buspar (buspirone) as an adjunct medication for anxiety. Depression and suicidal ideations responded robustly within 4-5 weeks of starting the esketamine treatment as assessed using the Hamilton Depression Rating Scale (HAM-D). We also noticed a significant improvement in her core BPD symptoms. Over the next one year with the same treatment, she had significant improvement in impulsivity and affective instability, and along with it, her HAM-D score gradually decreased over the next two years as shown in Figure 1.

Her self-harm attempts decreased in frequency from once every week to once every three months. The patient did not have any further cycles of restriction and binge eating, and she was able to maintain a stable weight. Her interpersonal relationships improved, and she was able to maintain a stable day job. She had spells of anxiety and some anger outbursts, but they decreased in frequency over time. She was able to utilize her coping skills during crises, as noticed by a decrease in further emergency department visits. She adequately handled the responsibility of taking care of her child, albeit with the help of her mother. Two years into treatment, the patient and her mother report around 70% improvement in her depression and anxiety with around 80% improvement in her behavioral symptoms. Her family reported the patient as being a “completely different person” post-esketamine treatment. Moreover, during the treatment period, she missed a few doses of esketamine due to the unavailability of medication, and this coincided with a marked increase in anger outbursts and self-harm attempts. This raised the question of whether the recurrent symptoms were from breakthrough depressive symptoms or her BPD symptoms. The patient is currently on maintenance esketamine treatment. She has received over 42 doses of esketamine over the last one and half years and remains compliant. It is notable that the patient’s compliance with treatment has been 100% as compared to previous treatments where her compliance has been poor.

## Discussion

Esketamine is a psychoactive drug that is fairly new to psychiatry as an FDA-approved treatment option. Ketamine, as an anesthetic agent, was discovered to have antidepressant properties in 1975. However, due to its property to induce a dissociative state and euphoria, ketamine soon became one of the most abused drugs [11,12]. The abuse potential meant that its use had to be judicious and cautious. Recently, ketamine has been gaining much more attraction as an agent to help relieve suicidality. This evidence was controversial due to the overlap of suicidality and depression in the study subjects. In a randomized control trial of 80 inpatients with an established diagnosis of MDD and suicidal ideations (SI), who were given a single infusion of 0.5 mg/kg ketamine as opposed to placebo, there was a notable decrease in SI partially independent of depressive symptoms at six weeks compared to the placebo group [10].

Esketamine is hypothesized to act through the (a) brain-derived neurotrophic factor/tropomyosin-related kinase B (BDNF-TrkB) system, (b) NMDA and AMPA receptor, (c) monoaminergic system, (d) opioid system, (e) low-voltage-sensitive T-type calcium channel, and (f) mTORC1 and ERK messenger system. Ketamine is also an atypical psychedelic [10]. Action at such varied targets has opened new arenas for ketamine, and studies have shown its potential use in eating disorders, obsessive-compulsive disorder, substance use disorder, pain management, and post-traumatic stress disorders. At the same time, concerns have been raised regarding the unregulated use of ketamine due to its role in opioid agonistic activities as a necessary mechanism of action for ketamine’s antidepressant effects [13,14].

It is noteworthy that ketamine has an action on various pathways that are considered defective in borderline patients. Glutamate plays a key role in personality traits such as impulsivity, aggression, and suicidal behavior [15]. Coccaro et al. reported direct correlations between cerebrospinal fluid (CSF) glutamate levels and composite measures of aggression, impulsivity, and impulsive aggression in healthy subjects and patients with personality disorders [16]. Glutamate-mediated neuroinflammation and HPA axis dysregulation are seen with BPD, early life trauma, and impulsivity [17,10]. Esketamine’s potential action on patients with BPD can be partially explained by its very well-documented effect on the glutamate receptor antagonism. Additionally, patients with stress-induced suicidal ideations, which are seen in borderline patients as well, are better responsive to ketamine [10].

Both physical and psychological pain converge into the opioid system, and low levels of endogenous opioids in BPD lead to chronic dysphoria, lack of sense of well-being, and a chronic feeling of emptiness. Self-harm attempts have been shown to increase opioid levels in patients with BPD [17]. Ketamine recalibrates and improves basal opioid levels, which can hypothetically improve symptoms of BPD [10].

Patients with borderline personality disorder have decreased connectivity between the prefrontal cortex (PFC) and the amygdala [18]. Ketamine improves connectivity in brain cortical areas such as the prefrontal cortex and amygdala and decreases diffuse mode network activity. This reduces negative self-attribution, which is hyperactive in patients with BPD and depression [10].

A recent case report discussed intravenous ketamine being detrimental in patients with borderline personality disorder and impulsivity. They noticed that after treatment with ketamine, the patient demonstrated increased disinhibition and an increase in suicidal ideations. These symptoms improved after ketamine therapy was discontinued [19]. This is contrary to our experience of managing a patient with BPD with esketamine. With BPD being such a heterogeneous disorder, it is possible that esketamine is advantageous in some subsets of BPD and not in others. Hence, there is a need for careful research in this area. The symptom resurgence in our patient on discontinuation of esketamine points toward the importance of maintenance treatment in such patients. The age group in which it can be used needs to be studied further as no studies have yet examined the impact of esketamine on the adolescent brain [20].

We recommend a trial of intranasal esketamine in patients with BPD with treatment-resistant MDD and frequent episodes of self-harm. Treatment with esketamine could potentially reduce the number of emergency room visits for impulsive suicide attempts and help reduce the life burden of BPD and its impact on family members. Other advantages may include optimization of medication management by reducing the number of medications often used by clinicians used to treat BPD. The two-hour observation period for esketamine treatment presents an opportunity to engage the patient in psychotherapy.

## Conclusions

With this case report, we consider the possibility that at least a subset of patients with BPD may benefit from esketamine, and this should be explored further. There is a need to delineate if these beneficial effects could be replicated in patients with BPD who do not have treatment-resistant depression. The specific characteristics of patients with BPD that respond to esketamine remain unknown at this time.
